# Gut Microbiota Approach—A New Strategy to Treat Parkinson’s Disease

**DOI:** 10.3389/fcimb.2020.570658

**Published:** 2020-10-22

**Authors:** Jing Liu, Fei Xu, Zhiyan Nie, Lei Shao

**Affiliations:** ^1^Department of Microbiology and Immunity, The College of Medical Technology, Shanghai University of Medicine & Health Sciences, Shanghai, China; ^2^Microbial Pharmacology Laboratory, Shanghai University of Medicine & Health Sciences, Shanghai, China; ^3^State Key Laboratory of New Drug and Pharmaceutical Process, Shanghai Institute of Pharmaceutical Industry, Shanghai, China

**Keywords:** Parkinson’s disease, dopamine, intestinal neuromodulation, brain-gut axis, gut microbiota

## Abstract

Parkinson’s disease (PD) is a progressive neurodegenerative disorder characterized by neuronal loss and dysfunction of dopaminergic neurons located in the substantia nigra, which contain a variety of misfolded α-synuclein (α-syn). Medications that increase or substitute for dopamine can be used for the treatment of PD. Recently, numerous studies have shown gut microbiota plays a crucial role in regulating and maintaining multiple aspects of host physiology including host metabolism and neurodevelopment. In this review article, the role of gut microbiota in the etiological mechanism of PD will be reviewed. Furthermore, we discussed current pharmaceutical medicine-based methods to prevent and treat PD, followed by describing specific strains that affect the host brain function through the gut-brain axis. We explained in detail how gut microbiota directly produces neurotransmitters or regulate the host biosynthesis of neurotransmitters. The neurotransmitters secreted by the intestinal lumen bacteria may induce epithelial cells to release molecules that, in turn, can regulate neural signaling in the enteric nervous system and subsequently control brain function and behavior through the brain-gut axis. Finally, we proved that the microbial regulation of the host neuronal system. Endogenous α-syn can be transmitted long distance and bidirectional between ENS and brain through the circulatory system which gives us a new option that the possibility of altering the community of gut microbiota in completely new medication option for treating PD.

## Introduction

Parkinson’s disease (PD), also known as tremor paralysis, is a common neurological degenerative disease in the elderly, characterized by the lesions of substantia nigra and striatum (Liepelt-Scarfone et al., 2013). Tremor, muscle rigidity, bradykinesia, and unstable posture are the main clinical symptoms of this disease. PD is associated with a variety of factors, including cerebrovascular disease (Haugarvoll et al., 2005), cerebral arteriosclerosis (Kummer et al., 2019), infections (Limphaibool et al., 2019), poisoning (Taba, 2017; Li et al., 2018), trauma, medications (Höllerhage, 2019), and genetic defects (Vila and Przedborski, 2004; Manzoni and Lewis, 2013). PD is the second most common neurodegenerative condition after Alzheimer’s disease which affects patients in the world. With the acceleration of the population aging process and decrease of physical functions of senile patients, the incidence and prevalence of PD have shown an increasing trend year by year (Nussbaum and Ellis, 2003; Hirsch et al., 2016). Approximately between 4.1 million and 4.6 million people are affected by PD in 2005 totaled, and it has been estimated that number will more than double by 2030 to between 8.7 million and 9.3 million in the most populous nations (Dorsey et al., 2007; Wang et al., 2020). The majority of people who get PD are over the age of 60, the incidence of PD among people over 60 is about one percent, men are more susceptible to PD than women at a ratio of about 3:2 (De Lau and Breteler, 2006; Dorsey and Bloem, 2018; Cerri et al., 2019).

The deposition of alpha-synuclein (α-syn) in neuronal cells could contribute to the development of PD. The α-syn is the most abundant protein constituent of Lewy bodies(LBs), which are generally described as round lamellar eosinophilic cytoplasmic inclusions (Braak et al., 2003a). LBs are the hallmark pathologic features of PD. Whether LBs are cytotoxic or cytoprotective to neuronal cells remains debatable. It could potentially be toxic since the number of cortical LBs positively correlated with the severity of symptoms of dementia in PD (Hurtig et al., 2000). However, in some cases, Lewy pathology is also found but without present parkinsonism (Parkkinen et al., 2008; Adler et al., 2010; Milber et al., 2012). Some studies have also suggested that α-syn aggregates might be protective (da Costa et al., 2000; Tanaka et al., 2004), while oligomers and pre-fibrillar α-syn are the toxic species responsible for neurodegeneration (Chen et al., 2009). The excessive accumulation of a-syn can enhance its toxicity and lead to the degeneration of DA (dopamine) neurons in the substantia nigra of the midbrain in PD, and the loss of neurons is associated with motor symptoms (Dickson et al., 2009). It is also possible rather than the neuronal loss, the presynaptic terminal failure may be the more critical pathogenic factor for motor symptoms of PD (Schulz-Schaeffer, 2010). Besides motor symptoms, PD is also associated with non-motor symptoms. In the early stage of PD, non-motor symptoms such as insomnia, impairment of smell (Shah et al., 2009) as well as gastrointestinal (GI) dysfunction (nausea, abnormal salivation, constipation, prolonged intestinal transit time etc.) (Cersosimo et al., 2013; Mulak and Bonaz, 2015) can be found. The typical movement-related symptoms, such as tremor, rigidity, bradykinesia, and postural instability are reported in the second stage of PD. In the final stage, the severe psychotic symptoms such as motor disorders and neuropsychiatric disturbances which including depression (Marsh, 2013; Parashar and Udayabanu, 2017; Guo et al., 2020; Park et al., 2020), dementia (Tsuang et al., 2013) can be observed in PD patients. The interesting phenomenon is that PD patients who suffer from GI symptoms can occur several years ahead of classic motor symptoms (Chen et al., 2015). The GI dysfunction caused by gut microbiota disorder which can initiate α-syn accumulation in the enteric nerve cell, causing concurrent mucosal inflammation and oxidative stress (Chen et al., 2019). So scientists give the hypothesis that PD may begin in the gastrointestinal tract and transfer to the brain through the gut-brain axis (Hawkes et al., 2007).

It has been shown that the intestinal microbiota and its metabolites can be involved in modulating a lot of GI functions, such as intestinal permeability (Frazier et al., 2011), mucosal immune function (Simrén et al., 2013), the motility (Cani et al., 2013) and sensitivity of the intestine (Valdez-Morales et al., 2013), as well as the activity in the ENS(enteric nervous system) (Forsythe and Kunze, 2013). The microbiota and its metabolites are also likely to modulate behaviors and brain processes, including stress responsiveness (Dinan and Cryan, 2012), emotional behavior (Foster and Neufeld, 2013), pain modulation, ingestive behavior (Cryan and Dinan, 2012), and brain biochemistry (Stilling et al., 2014). Therefore, altering the community of gut microbiota through prebiotics and antibiotics or fecal transplantation can give a new approach to treat PD due to gut microbiotas play a significant role in the neuropathogenesis of CNS (central nervous system) disorders.

## The Etiological Mechanism of PD

Aging is the most important risk factor for PD, and the biochemical changes caused by aging exacerbate these abnormalities in the brain of PD (Reeve et al., 2014). Dysfunction of DA neurons will cause a neuronal loss in the substantia nigra, which ultimately leads to inhibition of motor cortex neuron activation and function (Sulzer, 2007). In PD patients, motor symptoms are mainly related to the loss of DA neurons in the substantia nigra (Yamada et al., 2004). Moreover, neuropathological changes could be found in the autonomic nervous system, olfactory structures, the lower brainstem, and cerebral cortex (Dickson, 2012; Rey et al., 2016). Extrapyramidal pathology is associated with a wide range of non-motor symptoms, which is considered as an important feature of PD (Lim and Lang, 2010). It is reported that about 80% of PD patients have gastrointestinal dysfunction especially constipation (Frazzitta et al., 2019) and the GI dysfunction can occur several years before the onset of motor symptoms. Idiopathic constipation is one of the most substantial risk factors for PD (Poirier et al., 2016). For many years, people have understood that the environmental and genetic factors can cause loss of DA neurons in the substantia nigra, which has been dramatically expanded the understanding of the etiology of PD (Schapira and Jenner, 2011). Based on lots of experiment investigations, people have reached a consensus on the mechanism of cell death induced by toxins (Anselmi et al., 2018), while how the genetic defects lead to the loss of the neurons in PD is not clear. The neuronal cell death could be caused by apoptosis or autophagy. Mitochondrial dysfunction, oxidative stress, altered protein handling, and inflammatory could be involved in the neuronal cell death (Lin and Beal, 2006; Schapira and Jenner, 2011). Mitochondria play a critical role in cellular energy metabolism, mitochondrial dysfunction and LBs formation are vital to the pathogenesis of PD (Golpich et al., 2017).

As shown before, the formation of LBs is very important for the understanding of pathogenesis in PD. Lewy neurites are the elongated structures in dendritic or axonal compartments that are in the central and peripheral nervous systems (Volpicelli-Daley et al., 2014). Both LBs and Lewy neurites are mainly composed of filaments of misfolded α-syn protein (Spillantini et al., 1998; Goedert et al., 2013) The native conformation of α-syn is a soluble monomer that serves a pivotal role in synaptic transmission and enhances the transmitter release from the presynaptic vesicle (Burré, 2015). The α-syn protein is generally expressed in the CNS with a function of modifying the supply and release of DA to regulate neurotransmission in the brain (Longhena et al., 2019). The intermediate oligomeric protofibrillar form of α-syn has been suggested to be the most toxic species (Goldberg and Lansbury, 2000). Their accumulation at presynaptic terminals will affect the pivotal steps of neurotransmitter release (Bridi and Hirth, 2018). In PD patients, the progressive degeneration of DA neurons in the dense substantia nigra of the midbrain is the main pathological change of PD (Lindvall and Kokaia, 2009).

Damage in synaptic activity by α-syn microaggregation plays a key role in DA neurons degeneration (Calo et al., 2016). In healthy conditions, the correct organization of synaptic vesicle pools in a dopaminergic striatal terminal can be observed in the brain, monomer α-syn by regulating DA transporters can control preserved DA release and reuptake (Burré, 2015). In the prodromal phases of PD, high levels of α-syn microaggregate at synaptic terminals, this will alter the size of synaptic vesicle pools. The trafficking between the reserve and readily releasable pools will be impaired (Bridi and Hirth, 2018). Misfold α-syn can misregulate or redistribute proteins of the presynaptic Soluble NSF Attachment Protein Receptor (SNARE) complex, synaptic vesicles cluster, and their recycling was attenuated (Wang et al., 2014; Bellucci et al., 2017). Furthermore, α-syn overexpression reduces dopamine transporter (DAT) membrane content and reduces DA release (Vaughan and Foster, 2013). The presynaptic alterations impair neurotransmitter exocytosis and neuronal communication. Terminal loss and axonal or cell body degeneration not yet happen during this stage. In the early stages of PD, loss of neuronal connections at terminals could trigger axonal damage synaptic and axonal loss, the onset of symptoms is related to these changes (Caminiti et al., 2017). DAT binding decreased and partial Nigrosome-1 degenerated (Wang et al., 2017). Finally, in the advanced phases of PD, broad synaptic, axonal, and cell body degeneration can be detected concomitantly participate in disease progression. The degeneration of DA neurons in PD can be mediated by apoptosis. Two particular proteins have an essential function in the process of apoptosis: DRP1 promotes mitochondrial cytochrome C release, while the OPA1 inhibits cytochrome C release (Estaquier and Arnoult, 2007; Sheridan and Martin, 2010). When the balance between the two proteins is broken, a large number of cytochrome C are released, the cell death process will happen (Suen et al., 2008). Once apoptosis is activated, amoeba changes, cell membranes blister, cytoskeleton collapse, and cytoplasm condense will also happen. These will cause nuclear agglutination, chromosome agglutination or fragmentation, plasma membrane bleb and apoptotic body formation (Elmore, 2007). Apoptotic signals are transmitted to the mitochondria, causing the release of cytochrome C, which is located in the intermembrane of mitochondria, where it acts as an electron shuttle function in the respiratory chain (Li et al., 2000). Cytochrome C binds to APAF-1 and activates caspase 9, causing protein hydrolysis and eventually leading to neuronal apoptosis (Woo et al., 2003). Under normal physiological conditions, neuronal cells have a highly resistant ability to apoptosis in the late stage of mitosis. However, pathologic apoptosis can occur in nerve cells under some stimulus. In recent years, more and more researchers have realized the importance of abnormal apoptotic pathways in the pathogenesis of PD (Lama et al., 2020).

**Figure 1:**
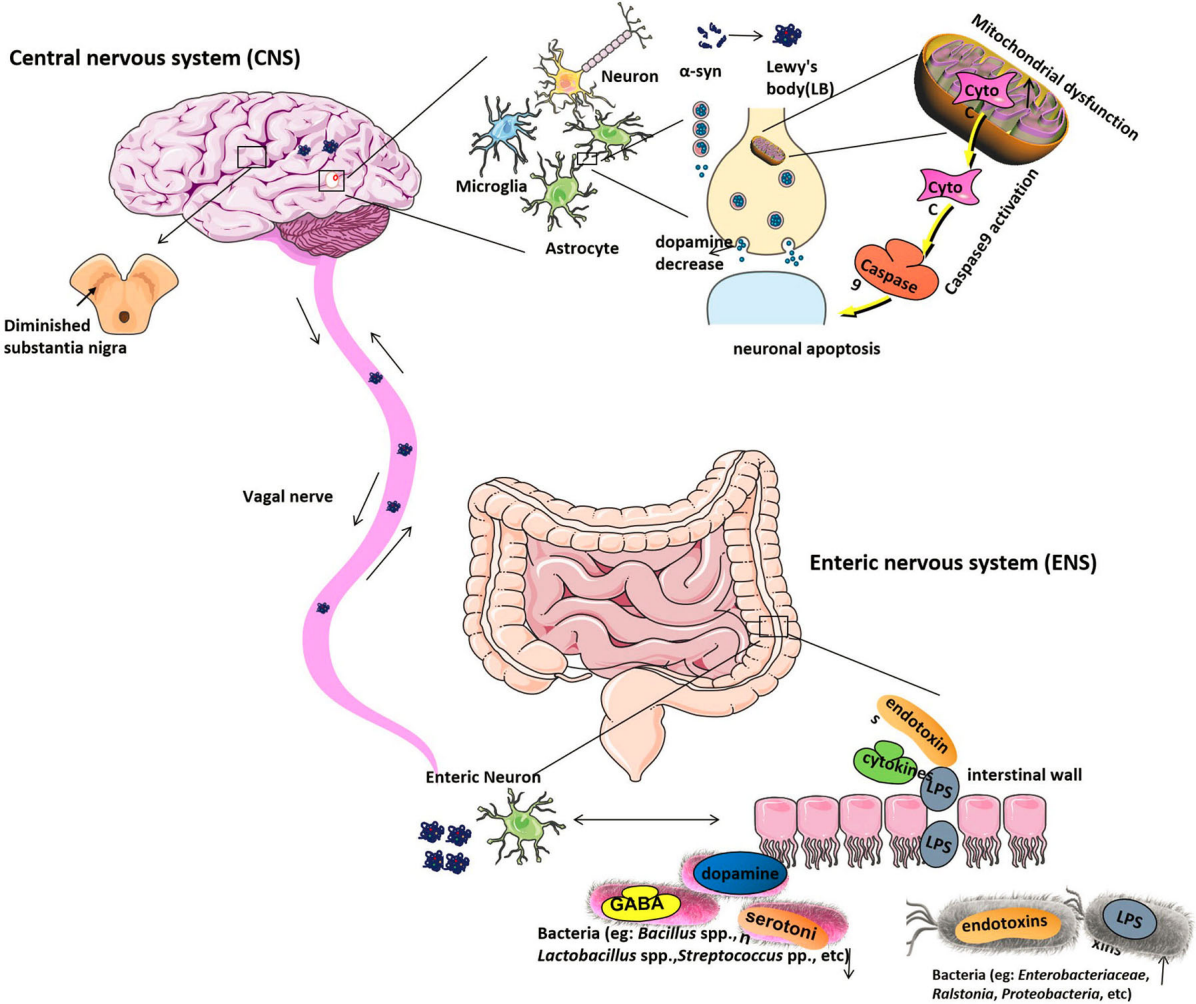
The etiological mechanism of Parkinson’s disease. Gut microbiota dysbiosis leads to increased intestinal permeability and systemic exposure of bacterial endotoxins, thereby initiating excess α-syn expression and supporting its misfolding to form LBs. The intestinal LBs from ENS will reach the CNS through the vagal nerve and eventually move to and damage the substantia nigra, which will lead to the appearance of the clinical symptoms of PD. The α-syn protein is generally expressed in the CNS with a function of modifying the supply and release of dopamine to regulate neurotransmission in the brain, while, in PD patients’ brains, α-syn protein is overexpressed and forms the LBs will cause dopamine release decreased. Moreover, LBs are the most toxic species in the brain. Mitochondrial dysfunction can be found under pathological conditions. Apoptotic signals are transmitted to the mitochondria, causing the release of Cyto C, which located in the intermembrane of mitochondria. Cyto C activates caspase9, causing protein hydrolysis and eventually leading to neuronal apoptosis. Under normal physiological conditions, neuronal cells have a highly resistant ability to apoptosis. However, when the conditions are pathological, their auto-apoptosis can occur abnormally and cause DA neurons degeneration. Bacteria, including *Enterobacteriaceae*, *Ralstonia*, *Proteobacteria*, *etc*. are increased in PD stool samples, which will raise the serum lipopolysaccharide (LPS) and other endotoxins concentration. And bacteria like *Bacillus* spp., *Lactobacillus* spp., *Streptococcus* spp. that can produce neurotransmitters such as gamma-aminobutyric acid (GABA), serotonin, and dopamine separately are decreased in PD stool samples. LB, Lewy’s body; CNS, central nervous system; ENS, Enteric nervous system; PD, Parkinson’s disease; Cyto C, Cytochrome C; DA, dopamine.

The researchers have found that α-syn not only can regulate the neurotransmission in the brain and also it can regulate the GI function. The α-syn forms and diffuse from the intestinal tract to the brain, supporting the hypothesis that PD pathogenesis may primarily function through the gut intestine, as shown in **Figure 1** (Holmqvist et al., 2014). It is reported that during the early stage of PD, the internal and external innervation of the GI tract, the dorsal motor nucleus of the vagus nerve (DMV) and the ENS of the vagus nerve were affected to various degrees by the intestine, suggesting that the PD pathogenesis observed in the gut were even earlier than the substantia nigra (Braak et al., 2003b). It has been proven that an unknown neurotropic pathogen initially damaged and disrupted the innervation of the GI tract and led to Lewy pathology of the intestine. The intestinal α-syn forms (including monomers, oligomers, and fibrils) reach the DMV through vagal innervation and eventually damage the substantia nigra, which lead to the appearance of the clinical symptoms of PD (Braak et al., 2006; Recasens and Dehay, 2014; Longhena et al., 2017) (**Figure 1**). According to this hyperthesis, the clinical pathology of PD can be found in the following three stages. In the early stage of PD, initial pathological α-syn appears in the olfactory bulb and DMV (Braak et al., 2003a; Forsyth et al., 2011). In the second stage of PD, substantia nigra be positive for immunoreactive α-syn inclusions (Repovš and Baddeley, 2006; Rey et al., 2016). In the final stage, as LBs reach the striatum and cerebral cortex, the severe psychotic symptoms of PD can be observed. It is reported that gut-initiated pathological processes in PD not only can be caused by a PD pathogen or environmental toxin, it also can be directly caused by gut microbiota disorder. Holmqvist proved that α-syn could be retrogradely transported from the intestinal wall to the brain by the experiment that the injection of α-syn into the intestinal wall of rats and track the transfer route (Holmqvist et al., 2014). Some researchers also found that α-syn can be transmitted *via* endocytosis to neighboring neurons by using *in vitro* and *in vivo* experiments (Hansen et al., 2011; Angot et al., 2012; Kim et al., 2019).

Since α-syn can be transmitted between brain and intestine which gives us a new option that we can modify the gut microbiota to alter the system of the intestine and reduce concurrent mucosal inflammation, eventually reduce the symptoms of PD. Nowadays, it is clear that certain bacteria are strain-specific that they can produce different essential neurotransmitters and specific neuromodulators. It is reported that several neurotransmitters such as gamma-aminobutyric acid (GABA), noradrenaline, serotonin, dopamine, and acetylcholine are produced by gut microbiota in human beings. For example, researchers reported that *Lactobacillus* spp., *Bifidobacterium* spp. (Y2) and *Streptococcus salivarius* subsp. thermophilus can produce GABA (Barrett et al., 2012; Pokusaeva et al., 2017). *Escherichia* spp., *Saccharomyces* spp., and *Bacillus* spp. can produce noradrenaline (Shishov et al., 2009; Rogers et al., 2016); *Streptococcu*s pp., *Candida* spp., *Enterococcus* spp., and *Escherichia* spp. produce serotonin (Özoğul, 2004; Shishov et al., 2009; Özoğul et al., 2012); *Bacillus* spp.(*Bacillus cereus*, *Bacillus mycoides, Bacillus subtilis*), *Escherichia coli* (K-12), *Hafnia alvei* (NCIMB, 11999), *Klebsiella pneumoniae* (NCIMB, 673), *Morganella morganii* (NCIMB, 10466), *Proteus vulgaris, Serratia marcescens*, and *Staphylococcus aureus* can produce dopamine (Özoğul, 2004; Shishov et al., 2009; Rogers et al., 2016); and *Lactobacillus* spp. produce acetylcholine (Rogers et al., 2016). The neurotransmitters secreted by the intestinal lumen bacteria may induce epithelial cells to release molecules that, in turn, can regulate neural signaling in the ENS and subsequently control brain function and behavior through the brain-gut axis. In some animal studies, various bacterial strains also mediate behavioral effects through the vagus nerve (Reid, 2019). Other important molecules produced in the colon by microbial fermentation of dietary fiber are short-chain fatty acids (SCFAs), such as butyrate, acetate, and propionate. SCFAs can regulate intestinal inflammation and immune function; they can be detected in the blood circulation system and also across the blood-brain barrier (BBB) *via* monocarboxylate transporters to affect the CNS system. SCFAs can promote microglia-mediated neuroinflammation (Sampson et al., 2016). For example, the administration of high doses of propionate in rats can cause neurasthenia (den Besten et al., 2013). Therefore, intestinal microorganisms and their products play important roles in improving the symptoms and pathogenesis of PD.

## The Current Treatments for PD

Current treatments for PD either increase/replace DA, or prevent the breakdown of DA, or prolong the action of levodopa to help control tremors (Isaacson and Hauser, 2009). Medications and surgery have been used to treat PD, but both have moderate side effects and often produce disappointing results. We will cover the current treatments related to PD and also discuss the side effects of different treatments (**Table 1**).

**Table 1:** Current treatments of Parkinson’s disease.

Group of treatment	Name of medicine	Pharmaceutical names	Mechanism of action	Drawbacks	Ref.
increase or substitute for dopamine	Carbidopa-levodopa^a^.	Lodosyn-levodopa	Levodopa converted to dopamine, carbidopa protect levodopa breakdown	Lightheadedness, nausea, dyskinesia	(Kell et al., 2017; Haddad et al., 2018; Lau et al., 2018)
	Duopa therapy^a^.	Duopa	Delivers the medicine in the gel, reduces motion fluctuations and movement disorders	The tube fall out, and infections, blockage in the tube	(Chaudhuri et al., 2016; LeWitt, 2016)
	Dopamine agonists^a^.	Requip, Mirapex, Neupro	Similar effects as dopamine	Hallucinations, sleepiness, and compulsive behaviors	(Pahwa and Lyons, 2009; Kulisevsky and Pagonabarraga, 2010; Yu and Fernandez, 2017)
		Apomorphine	Intermittent subcutaneous injections treat the motor symptoms of PD	Hallucinations, sleepiness, and compulsive behaviors	(Patel et al, 2017; Antonini and Nitu, 2018)
	MAO B inhibitors^a^.	rasagiline, safinamide, selegiline,	Prevent the breakdown of brain dopamine	Nausea, insomnia,	(Dezsi and Vecsei, 2017; Binde et al., 2018; Szökő et al., 2018)
	COMT inhibitors^b^	Comtan, Tasmar	Block the enzyme that breaks down dopamine;	Risk of serious liver damage, diarrhea, dyskinesia	(Schlesinger and Korczyn, 2016; Katsaiti and Nixon, 2018; Silva et al., 2020)
	Anticholinerg^b^	Cogentin, trihexyphenid-yl	Used as monotherapy or combination regimen, they work better on tremors	Impaired memory, hallucinations, dry mouth, and impaired urination.	(Nishtala et al., 2016; Mishima et al., 2018; Morrow et al., 2018; Hong et al., 2019)
	Amantadine^b^	Amantadine	short-term relief of mild symptoms, control involuntary movements	Ankle swelling, skin purple mottling, or hallucinations	(Wolf et al., 2010; Kim et al., 2018)
	Creatine^b^	Creatine	Energy compound that exerts neuroprotective effects	Weight gain, impairment of renal function	(Xiao et al., 2014; Duarte-Silva et al., 2018; Marques and Wyse, 2019)
Surgical procedures	Deep brain stimulation^b^	DBS	Send electrical pulses to the patient’s brain and reduces the symptoms of PD	Infection, brain hemorrhage or stroke.	(Follett et al., 2010; Lee et al., 2018)
Gene therapy^c^	Gene therapy	GAD, GABA	Alter local neurotransmitters or neurotrophic factors in the basal	Clinical results have been less encouraging	(Bartus et al., 2014)
Immunotherapy^c^	Immunotherapy	α-syn immunotherapies	Using antibodies against misfolded α-synuclein	Induction of Th17 cell-mediated inflammatory autoimmunity,	(George and Brundin, 2015)
Cell transplantation^c^	Embryonic stem cells	Fetal mesencephalic tissue, stem cell	Introducing new dopamine cells into the brain of PD	Unacceptable graft-induced dyskinesia	(Normile, 2018)

a Standard therapeutic agents.

b Alternative therapeutic agents.

c Therapeutical agents under investigation.

### Medications Increase or Substitute for Dopamine

Since DA cannot enter into the brain, it cannot be given directly to treat PD. Medications increase or substitute for DA can be used to treat the PD.

#### Carbidopa-Levodopa

Levodopa can pass into the brain and be converted to DA to treat PD (Haddad et al., 2018) and carbidopa can prevent levodopa breakdown. Side effects may happen, including lightheadedness (orthostatic hypotension) (Lau et al., 2018) or nausea (Kell et al., 2017). As the disease progresses after years, the effect of levodopa becomes less stable, and a tendency to wane and dyskinesia may appear in PD (Tran et al., 2018).

#### Duopa Therapy

Duopa is a form of carbidopa/levodopa delivered in gel form. It delivers the medication directly to the small intestine in the gel form through a feeding tube (LeWitt, 2016). A pump slowly and consistently delivers Duopa to the intestine through the tube. This procedure allows the medicine to be absorbed smoothly and reduces motion fluctuations and movement disorders (Chaudhuri et al., 2016). The Duopa therapy has the risks that the tube may fall out or infections may happen at the infusion site or a blockage occurring in the tube.

#### Dopamine Agonists

DA agonists mimic DA effects in the brain, the effective time is longer than levodopa (Grall-Bronnec et al., 2018). Short-acting injectable DA agonists such as Requip, Mirapex, and Neupro can be used for quick relief in PD treatment (Pahwa and Lyons, 2009; Kulisevsky and Pagonabarraga, 2010; Yu and Fernandez, 2017). Apomorphine is a DA agonist that can be delivered by intermittent subcutaneous injections to treat the fluctuations in motor symptoms of PD (Antonini and Nitu, 2018). Side effects including hallucinations, sleepiness, and compulsive behaviors can be found (Patel et al, 2017).

#### MAO B Inhibitors

The brain enzyme monoamine oxidase B (MAO B) metabolizes the brain DA (Tabakman et al., 2004). MAO B inhibitors can prevent the breakdown of brain DA by inhibiting MAO B enzyme activities (Finberg, 2019). These MAO B inhibitors include rasagiline, safinamide, and selegiline (Deshwal et al., 2017; Dezsi and Vecsei, 2017; Binde et al., 2018; Szökő et al., 2018). Side effects including nausea or insomnia may happen (Dezsi and Vecsei, 2017).

#### Catechol O-methyltransferase (COMT) Inhibitors

COMT inhibitors mildly prolong the effect of levodopa by blocking an enzyme that can break down DA (Schlesinger and Korczyn, 2016; Katsaiti and Nixon, 2018). The medication from this class mainly includes Comtan and Tasmar (Olanow and Watkins, 2007; Lees, 2008). This medicine has a risk of serious liver damage and liver failure, other side effects include diarrhea or increased risk of dyskinesia (Silva et al., 2020).

#### Anticholinergics

Anticholinergics including Cogentin and trihexyphenidyl were used to control the tremor associated with PD (Olanow et al., 2001; Nishtala et al., 2016; Mishima et al., 2018). It is reported that they work better on tremors than on other PD characteristics (Lang and Lees, 2002). They are common side effects such as impaired memory, hallucinations, confusion, constipation, dry mouth, and impaired urination (Morrow et al., 2018; Hong et al., 2019).

#### Amantadine

It can provide short-term relief of mild symptoms. It can be used during the later stages of PD by giving together with carbidopa-levodopa therapy to control involuntary movements (Wolf et al., 2010). Side effects of amantadine may include ankle swelling, skin purple mottling, or hallucinations (Kim et al., 2018).

#### Creatine

Creatine is an energy compound that exerts neuroprotective effects in animal models of PD (Duarte‐Silva et al., 2018; Marques and Wyse, 2019). It also acts as an antioxidant protected against the loss of both Nissl and tyrosine hydroxylase in the substantia nigra (Xiao et al., 2014). Weight gain is the most common side effect of creatine, impairment of renal function can also be found (Bender et al., 2008).

### Surgical Procedures

#### Deep Brain Stimulation

DBS is offered to people with advanced PD (Lee et al., 2018). DBS stabilizes medication fluctuations, reduces or prevents dyskinesias, reduces tremors and stiffness, and improves movement slowness. In deep brain stimulation (DBS), a surgeon first implants electrodes into a specific part of the patients’ brain. Then the electrodes are connected to a generator implanted in the chest near the patient’s clavicle (Follett et al., 2010). Risks such as infections, brain hemorrhage, or stroke may happen.

### Gene Therapy

A lot of PD gene therapy clinical trials aim to alter local neurotransmitters or neurotrophic factors in the basal. Although these trials show that gene therapy can be safely delivered to the brain and induce specific neuronal protein expression, the clinical results have been less encouraging (Bartus et al., 2014).

### Immunotherapy

Immunotherapy targeted mainly using antibodies against misfolded α-syn (George and Brundin, 2015). Previous studies have tried to remove α-syn from extracellular space, thereby reducing the progressive deposition of α-syn aggregates throughout the brain (Masliah et al., 2005; Masliah et al., 2011). A possible side effect of immunotherapy is Th17 cell-mediated inflammatory autoimmunity involving in neurodegenerative neuritis (Reynolds et al., 2010).

### Cell Transplantation

Introducing new DA cells into the brain may help replace what is lost in PD. To date, there have been cell transplantation clinical trials using autologous and nonautologous cells, including the use of the human embryonic stem cells (ESCs) and induced pluripotent stem cells (iPS) (Parmar et al., 2020). The Japanese scientists have injected dopaminergic progenitor cells directly into an area of the brain associated with neural degeneration in PD in 2018 (Normile, 2018). The main challenge has been unacceptable graft-induced dyskinesia (Piquet et al., 2012).

## Microbiota-Targeted Intervention Strategies to Manage PD

Nowadays, it has been estimated that the human intestinal tract harbors a diverse and complex microbial community which plays an important role in many aspects of host physiology, including nervous system development and human neurodegenerative diseases (Thomas et al., 2017; Ceppa et al., 2020). The intestinal flora is currently considered a key regulator of a smooth two-way dialogue between the intestine and the brain (gut-brain axis). This fact provides a promising opportunity for preventing or treating neuropsychiatric conditions in PD. The relationship between gut flora and the brain can be traced back to brain development. After the fetus is born, the microorganisms obtained from the mother and the environment colonize the fetus’s intestine and play a critical role in brain development (Codagnone et al., 2019). The functions of gut microbiota include participation in the synthesis of multiple vitamins and fatty acids, and regulation of brain-derived neurotrophic factor (BDNF), synaptophysin, post-synaptic density protein 95 (PSD-95) (Sudo et al., 2004). A recent study has shown that injections of LBs from PD into the striatum of baboons or the intestine could induce the damage of the nigrostriatal pathway and the pathological changes of the ENS. No pathological damage of α-syn was detected in the vagus nerve and the DMV, suggesting that DMV may not be the pathologic transmission route of α-syn. The levels of α-syn in the blood of baboons injected were increased, which was positively correlated with the levels of α-syn in ENS. Endogenous α-syn may be transmitted long-distance and bidirectional between ENS and the brain through the circulatory system (Arotcarena et al., 2020).

It has been found in sterile animal research that the intestinal flora is necessary for the healthy development of the nervous system, and the nervous system function is challenging to mature sterile animals because of the lack of intestinal flora (Luczynski et al., 2016). It was found that compared with normal mice, the expression of BDNF in the cerebral cortex and hippocampus of sterile mice was significantly reduced (Arentsen et al., 2015). And sterile mice were more likely to show anxiety and less activity performance. After transplanting the healthy intestinal flora to sterile mice, it was showed increased activity and decreased anxiety in mice, and the 5-HT content of norepinephrine, DA, and terminal brain striatum also significantly increased (De Theije et al., 2014). The anxious behaviors and activities of SPF mice indicated that the colonization rate of the GI flora during colonization could affect the corresponding excitatory neuron cell signaling mechanism to some extent.

### The Association between Gut Microbiota Alteration and PD

There are associations between the composition of gut microbiota alteration and multiple prodromal markers of PD. Several studies have proven that certain bacterial taxa can be used as biomarkers or even drug targets for PD. A study showed that gut microbiota dysbiosis was observed in the PD compared to the healthy group. OTUs include *Proteus* sp.*, Bilophila* sp., and *Roseburia* sp., were increased with PD microbiomes and members of families *Lachnospiraceae, Rikenellaceae*, and *Peptostreptococcaceae*, as well as *Butyricicoccus* sp. were decreased (Scheperjans et al., 2015). Another study was supporting that compared to the healthy control, the levels of *Lactobacillus*, *Prevotellaceae*, *Peptostreptococcus*, and *Butyricicoccus* spp. are lower and the levels of *Proteus* and *Enterobacter* spp. are higher (Sampson et al., 2016). A recent clinical trial including 666 elderly subjects was done to analyze the association between PD risk factors and prodromal symptoms markers with the composition of gut microbiota. The physical activity, occupation-related solvent exposure, and constipation were associated with the α-diversity of gut microbiota, and the physical activity, gender, constipation, REM sleep behavior disorder (RBD), as well as smoking, are associated with β-diversity of gut microbiota, the age and uric acid-lowering drugs are associated with both α- and β-diversity of gut microbiota. Physical inactivity and constipation in individuals were highest common with *Firmicutes*-enriched enterotype, while constipation is the least common among individuals with *Prevotella*-enriched enterotype (Heinzel et al., 2020). Another study showed *Ralstonia*, *Proteobacteria*, *Enteococcaceae* concentration in the mucosa of PD patients increased. These bacteria have pro-inflammatory cytokine producing function. Anti-inflammatory bacteria including *Blautia*, *Coprococcus*, *Roseburia*, and *Faecalibacterium* in the stool samples of PD patients decreased (Keshavarzian et al., 2015) The LPS (lipopolysaccharide) biosynthesis genes were also reported significantly increased in the PD fecal samples (Keshavarzian et al., 2015). *Helicobacter pylori* infection is also related to trigger the pathogenesis in PD (Çamcı and Oğuz, 2016). A two-year following study showed that low counts of *Bacteroides fragilis* were related to worsening of motivation/activeness and *Bifidobacterium* was associated with hallucinations/delusions (Minato et al., 2017).

A study used the microbiome-wide association study (MWAS) in two large datasets to specify the gut microbiota alteration in PD. Cluster 1 which was composed of opportunistic pathogens including *Porphyromonas*, *Corynebacterium*, *Prevotella, Porphyromonas*, and *Corynebacterium* were increased in PD. Genera in Cluster 2 including (*Oscillospira*, *Lachnospiraceae_UCG-004*, *Lachnospiraceae_ND3007_group*) and (*Agathobacter*, *Butyricicoccus*, *Blautia*, *Faecalibacterium*, *Lachnospira*, *Fusicatenibacter*, *Roseburia*) were reduced in PD. Most increased groups belong to *Ruminococcacea*e and *Lachnospiraceae* families which are already known as SCFAs producing bacteria. *Lactobacillus* and *Bifidobacteria* increased in PD in cluster 3. The genera in cluster 3 were probiotics with carbohydrate-metabolizing and possible of becoming opportunistic pathogens and immunogenic (Wallen et al., 2020). Nishiwaki et al. use a meta-analysis method compared 223 PD patients with 137 health controls and give a conclusion that genera *Akkermansia*, *Catabacter*, and families *Akkermansiaceae* were elevated, while *Roseburia*, *Faecalibacterium*, and *Lachnospiraceae ND3007* group were decreased in PD (Nishiwaki et al., 2020). When the dietary fibers defected, *Akkermansia muciniphila* can degrade the gut mucus layer and enhance enteric pathogen infection risk (Desai et al., 2016). Abundance *Akkermansia* can increase the permeability of intestine which exposes the intestinal neural plexus to an oxidative or toxic environment, and this may lead to α-syn ﬁbrils aggregate in the intestine. *Faecalibacterium* and *Roseburia* decreased in PD may provoke intestinal inﬂammatory, these two genera are butyrate-producing bacteria and butyrate belongs to SCFAs can induce anti-inﬂammatory cytokines gene expression by inhibiting histone deacetylase (Sokol et al., 2008; Canani et al., 2011). Cirstea also proved the intestinal function of PD is related to gut microbiota composition and metabolism (Cirstea et al., 2020). The microbiota composition of fecal samples as well as serum metabolomics were analyzed from 197 PD patients and 103 controls. There is a higher abundance of *Christensenellaceae*, *Desulfovibrionaceae*, *Biﬁdobacterium*, *Bilophila*, *Collinsella*, *Akkermansia* and lower abundance of *Lachnospiraceae*, *Roseburia*, *Faecalibacterium* in PD. The microbiota in PD showed reduced carbohydrate fermentation and low butyrate synthesis capacity, while the proteolytic fermentation and deleterious amino acid metabolites (p-cresol and phenylacetylglutamine) production were increased. The interesting phenomenon is that butyrate-producing bacteria were negatively associated with stool ﬁrmness since butyrate can regulate intestinal serotonin biosynthesis and improve the motility of colonic (Vincent et al., 2018; Cirstea et al., 2020). The SCFAs concentrations were significantly reduced in PD fecal samples. The *Bacteroidetes* (phylum) and *Prevotellaceae* (family) were reduced, and *Enterobacteriaceae* increased in PD. SCFAs may induce ENS alterations and dysmotility of gastrointestinal in PD (Unger et al., 2016). From all these studies, we could conclude that the opportunistic pathogens were increased, while potential benefit bacteria were reduced: *Prevotellace* decreased and *Enterobacteriaceae* increased in PD. The microbiome changed in PD is shown in **Table 2**.

**Table 2:** Alterations of gut microbiota compositions associated with Parkinson’s disease.

Comparison^a^	Microbiota	Sample	Mechanism	Ref.
PD patients vs Healthy control	*Proteus* sp.↑ *Bilophila* sp.↑*and Roseburia* sp.↑*Lachnospiraceae*↓*Rikenellaceae*↓ *Peptostreptococcaceae*↓*Butyricicoccus* sp. ↓	Stool	SCFA-producing families decrease	(Scheperjans et al., 2015)
PD patients vs Healthy control	*Lactobacillus*↓*Prevotellaceae*↓ *Peptostreptococcus*↓ *Butyricicoccus* spp.↓*Proteus*↑ *Enterobacter* spp.↑	Stool	Decreased *Prevotellace* lead to increased intestinal permeability, systemic exposure of bacterial endotoxins	(Sampson et al., 2016)
PD patients vs Healthy control	*Firmicutes* ↓ *Prevotella* ↑ *Faecalibacterium* ↓	Stool	SCFA-producing taxon decrease	(Heinzel et al., 2020)
PD patients vs Healthy control	*Ralstonia*↑*Proteobacteria*↑ *Enteococcaceae*↑*Blautia*, *Coprococcus*↓, *Roseburia*↓and *Faecalibacterium*↓	Stool	Pro-inflammatory cytokine producing bacteria increased, anti-inflammatory bacteria decreased	(Keshavarzian et al., 2015)
PD patients vs Healthy control	*Helicobacter pylori*↑	Stool	*Helicobacter pylori* is a triggering factor in PD pathogenesis	(Çamcı and Oğuz, 2016)
PD patients vs Healthy control	*Bacteroides fragilis* ↓ *Bifidobacterium*↓	Stool	*Bacteroides fragilis* were related with worsening of motivation/activeness and *Bifidobacterium* was associated to hallucinations/delusions	(Minato et al., 2017)
PD patients vs Healthy control	*Porphyromonas*↑*Corynebacterium*↑*, Prevotella*, ↑*Porphyromonas*, ↑ *Ruminococcaceae* ↓*Lachnospiraceae*↓ *Lactobacillus* ↑*Bifidobacteria*↑	Stool	Opportunistic pathogens were increased, SCFAs producing bacteria reduced, probiotics with carbohydrate-metabolizing increased	(Wallen et al., 2020)
PD patients vs Healthy control	*Akkermansia*↑ *Catabacter* ↑*Akkermansiaceae*↑*Roseburia*, ↓*Faecalibacterium*↓ *Lachnospiraceae*↓	Stool	*Akkermansia* can increase the permeability of intestine, lead to α-syn fibrils aggregate in intestine butyrate producing bacteria decrease	(Nishiwaki et al., 2020)
PD patients vs Healthy control	*Christensenellaceae*, ↑*Desulfovibrionaceae*↑*Bifidobacterium*↑*Bilophila*↑*Akkermansia*↑ *Lachnospiraceae*↓*Roseburia*↓*, Faecalibacterium*↓	Stool, serum	Carbohydrate fermentation reduced, low butyrate synthesis capacity proteolytic fermentation and deleterious amino acid metabolites production increased	(Cirstea et al., 2020)
PD patients vs Healthy control	*Bacteroidetes* ↓*Prevotellaceae* ↓*Enterobacteriaceae*↑	Stool	SCFAs may induce ENS alterations and dysmotility of gastrointestinal in PD	(Unger et al., 2016)
PD patients vs Healthy control	*Lactobacillus casei shirota* ↓*staphylococci* ↑	Stool	*Lactobacillus casei* shirota can improve the bowel movement by decreasing the number of *staphylococci* in PD patients	(Cassani et al., 2011)
PD patients vs Healthy control	*Bacillus* spp.↓	Stool	convert L-tyrosine to L-DOPA	(Surwase and Jadhav, 2011)
PD mice vs Healthy control	*Proteobacteria*↑ *Turicibacterales*↑*Enterobacteriales*↑ *Firmicutes*↓ *Clostridiales*↓	Stool	Fecal SCFAs concentration decrease, increase DA and 5-HT levels, reduce activation of microglia and astrocytes	(Sun et al., 2018)

a A comparison of condition A vs condition B; ↑, increase in condition A related to condition B; ↓, decrease in condition A related to condition B.

The interplay between α‐syn and gut microbiota attracts a lot of researchers’ interest. A previous study has already confirmed that increased expression of α‐syn in the substantia nigra can cause pathology of CNS, including motor and cognitive functions impaired (Crowley et al., 2018). Nigral overexpression of α‐syn reduced neuronal number in myenteric submucosal plexus, increased glial expression in the myenteric plexus, modulated myenteric and submucosal TH (tyrosine hydroxylase) intensity, alter gut microbiota as well as bile acid composition (O’Donovan et al., 2020). Plexus neuronal loss can affect epithelial barrier integrity, secretomotor functions and immune cell migration, which can increase the permeability of intestine and GI inflammation. Potentially beneficial bacteria *Faecalibacterium prausnitzii*, *Prevotellaceae*, and *Lactobacillaceae* were reduced in PD, the abundance *of Enterobacteriaceae* was increased. Nigral overexpression of α‐syn increased the level of fecal free bile acids. The distributions of bile acid indicate the liver synthesis increased or transporter deficiencies and reabsorption of bile acid in the small intestine also reduced. CA (cholic acid) and DCA (deoxycholic acid) have a role in cognitive decline (MahmoudianDehkordi et al., 2019). Increased DCA levels can inhibit the motility of GI. The DCA level were significant positive correlations with *Ruminococcaceae* and significantly negatively with *Lactobacillus* (O’Donovan et al., 2020). Gorecki used a Thy1-αSyn PD mice model, and found LPS can induce inflammation and alter the distribution of tight junction proteins. The mucin-degrading *Verrucomicrobiae* and LPS-producing *Gammaproteobacteria* were increased in PD patients. LPS administration leads to the increasing of intestinal permeability, motor impairment, nigral α‐syn aggregation, dopaminergic neuronal loss and reduction in striatal dopamine. So LPS-producing bacteria increasing can change the gut environment and trigger the pathogenesis of PD by α‐syn aggregation (Gorecki et al., 2019). LPS or inflammatory endotoxin modulate α‐syn amyloidogenesis by the formation of intermediate nucleating species. LPS-binding structural motif interacts with soluble monomer stabilizes the α-helical intermediates in the α-syn aggregation pathway. By saturation, transfer LPS can mediate the nucleation probe. Finally, the nucleating intermediates mediated by LPS mature into divergent fibrillary forms. LPS-induced can alter the backbone motility of α‐syn, modulate α-syn aggregation, and increase LPS-α‐syn fibril formation which is toxic in PD (Bhattacharyya et al., 2019). Thus gut microbiota plays an important role in the pathology of PD.

### Microbial Regulation of Host Neuronal System

The *Prevotellaceace* family members are important mediators of host nutrition. They can ferment complex polysaccharides to product SCFAs and modify bile acids through dietary metabolism. (Arumugam et al., 2011). The SCFAs receptors 2 (FFAR2) and 3 (FFAR3) were found expressed in the ENS, portal nerve and sensory ganglia system. The microbiota metabolites can directly function to sensory neurons or can signal to neurons *via* intermediate interactions with enteroendocrine or epithelial cells and regulate the host behavior (Egerod et al., 2018). The *Prevotellaceae* decreased which lead to intestinal permeability increased and bacterial endotoxins exposure (**Figure 1**), thereby initiating or retaining excess α-syn expression in the colon and supporting its misfolding (Sarkar and Banerjee, 2019). The increased *Enterobacteriaceae* in PD can raise the serum LPS concentration and the relative abundance of the *Enterobacter* spp. is also positively correlated with the severity of posture instability and gait difficulties of PD patients (Lin et al., 2019). LPS is derived from the gram-negative bacteria cell walls and crosses the intestinal wall then enter into the bloodstream and result in intestinal epithelial barrier disruption (Guo et al., 2013). LPS in the bloodstream may induce systemic inflammation (Tufekci et al., 2011), LPS as well as inflammatory cytokines like tumor necrosis factor (TNF-α), interleukin (IL)-1β, and IL-6 can disrupt BBB and promotes α-syn misfolding (Block et al., 2007), which lead to the destruction of DA neurons in the substantia nigra (Rite et al., 2007). Thus the overgrowth of *Enterobacter* spp. are correlated with the progression of PD (Mulak and Bonaz, 2015; Nair et al., 2018).

The approach that gut microbial interventions can be used to treat PD is supported by the fact that the gut microbiota can directly produce neurotransmitters or regulate the host biosynthesis of neurotransmitters (Bested et al., 2013), as shown in **Table 3**. But what are the functions of the neurotransmitters in gut microbiota and how they function on the host neuronal system is still not clear. A recent study shows that the *Bacteroides fragilis* can synthesis GABA and supported the KLE1738 growth, which means the GABA may be served as the growth substrate for KLE1738 (Strandwitz et al., 2019). Another study showed the 5-HT can increase the colonization rate of *Turicibacter sanguinis*, suggesting the role of neurotransmitter modulate bacterial colonization in the gut (Fung et al., 2019). Almost half of the host dopamine is produced by gut microbiota and up to 60% of colonic and blood 5-HT levels are biosynthesis by gut microbiota (Yano et al., 2015). Microbiota modulates the 5-HT activates in the intestine and increase the motility of GI. Microbiota also can regulate the local 5-HT to impact the central nervous system, an increasing number of researches report that the microbiome affects the host neuronal system (Sgritta et al., 2019). In the future, the microbiota specific functions on the neurological disorders and use the microbiota as the potential medical treatment for PD are needed to assess.

**Table 3:** Functions of gut microbiota on Parkinson’s disease.

Functions	Bacterial species	Functional substance	Mechanism of action	Ref.
Neurotransmitters secretion	*Lactobacillus* spp.*, Bifidobacterium* spp. *(Y2) Streptococcus salivarius subsp. thermophilus*	GABA	GABA secretion, regulate neural signaling in the enteric nervous system, control the growth of hormone secretion, control brain function and behavior	(Barrett et al., 2012; Pokusaeva et al., 2017)
	*Escherichia* spp.*, Saccharomyces* spp. and *Bacillus* spp.	Noradrenaline	Noradrenaline secretion, regulate neural signaling in the enteric nervous system	(Shishov et al., 2009; Rogers et al., 2016)
	*Streptococcu*s pp., *Candida* spp., *Enterococcus* spp. and *Escherichia* spp.	Serotonin	Serotonin secretion, regulate neural signaling in the enteric nervous system	(Özoğul, 2004; Shishov et al., 2009; Özoğul et al., 2012)
	*Bacillus* spp., *E. coli*, *Hafnia alvei*, *Proteus vulgaris*, *Serratia marcescens*	Dopamine	Convert l-tyrosine to L-DOPA, regulate neural signaling in the enteric nervous system	(Özoğul, 2004; Shishov et al., 2009; Rogers et al., 2016)
	*Lactobacillus* spp.	Acetylcholine	Acetylcholine secretion, induce epithelial cells to release molecules can regulate neural signaling in the enteric nervous system	(Reid, 2019; Rogers et al., 2016)
Fermentation of dietary fiber	*Prevotellaceae*	Butyrate, acetate and propionate	Production of mucin and SCFAs, decreased SCFAs lead to increased intestinal permeability, exposure endotoxins, initiate excess α-syn expression and misfolding	(Sampson et al., 2016; Sarkar and Banerjee, 2019)
Rise serum lipopolysaccharide(LPS)	*Enterobacteriaceae Gammaproteobacteria*	LPS	Rise the serum LPS population, induce systemic inflammation, promotes α-synuclein deposition, increase LPS-α‐syn fibril formation	(Guo et al., 2013; Lin et al., 2019; Gorecki et al., 2019; Bhattacharyya et al., 2019)
Induce inflammatory responses	*Ralstonia, Proteobacteria, Enteococcaceae*	Pro-inflammatory cytokine	Increase of pro-inflammatory cytokine	(Keshavarzian et al., 2015)
Anti-inflammatory	*Blautia, Coprococcus,and Roseburia and Faecalibacterium*	Butyrate	The butyrate-producing bacteria such as *Blautia, Coprococcus,Roseburia* and *Faecalibacterium* decreased which have anti-inflammatory function	(Keshavarzian et al., 2015; Qiao et al., 2020)
Triggering factor in PD pathogenesis	*Helicobacter pylori*		Triggering factor in PD pathogenesis	(Keshavarzian et al., 2015; Çamcı and Oğuz, 2016)
Worsening of motivation	*Bacteroides fragilis,Bifidobacterium*		Low counts of *Bacteroides fragilis* related with worsening of motivation/activeness and *Bifidobacterium* decreasing related with hallucinations/delusions	(Minato et al., 2017)
Improve the bowel movement	*Lactobacillus casei* shirota		Improve the bowel movement, the number of fecal *staphylococci* was decreased	(Cassani et al., 2011)
Increase bile acid	*Ruminococcaceae Lactobacillus*	CA and DCA	Have a role in cognitive decline	(MahmoudianDehkordi et al., 2019; O’Donovan et al., 2020);
Converting levodopa to dopamine	*Enterococcus, Lactobacillus Staphylococcus*	Tyrosine decraboxylase (TDC)	TDC in genome of bacterias, have the ability of converting levodopa to dopamine	(Zoetendal et al., 2012; van Kessel et al., 2019)
Neuroprotective effects	*B. animalis lactis*, *L. rhamnosus GG L. acidophilus*	Butyrate	Induce BDNF and glial cell line-derived neurotrophic factor (GDNF) upregulated	(Srivastav et al., 2019)

### Gut Microbiota Approach to Treat PD

#### Antibiotics

Koutzoumis et al. test broad-spectrum antibiotics function on oxidopamine injected rat PD model and found 90% of microbial richness was reduced. The level of *Firmicutes* was reduced, while *Proteobacteria*, *Verrucomicrobia*, *Bacteroidetes*, and *Cyanobacteria* increased. Antibiotics treatment can decrease striatum IL-1b and TNF-α levels, protect dopaminergic neuron cell loss and alleviate motor deficits in the PD rodent model (Pu et al., 2019; Koutzoumis et al., 2020).

#### Probiotics

Probiotics treatment has been proven to be a useful method to improve the PD. Probiotics strain *bifidobacteria* and *lactobacilli* have been reported to reverse PD conditions. The regular intake of fermented milk beverages containing the probiotic *Lactobacillus casei* shirota has been shown to improve the bowel movement and inhibit *staphylococci* growth in PD patients (Cassani et al., 2011). Probiotic bacterium *Bacillus* spp. can convert L-tyrosine to L-DOPA, which can supply the lost dopamine of PD (Surwase and Jadhav, 2011). Some bacteria in the gut can convert levodopa to dopamine through tyrosine decarboxylases(TDC). TDC has been identified in the genome of more than 50 *Enterococcus* strains, several *Lactobacillus* and *Staphylococcus*, which are potential probiotics of the small intestine (Zoetendal et al., 2012; van Kessel et al., 2019). Mediterranean diet (MeDi) which contains a large quantity of *Lactobacilli* is shown have effective in preventing Alzheimer’s disease, several clinical studies also show that higher MeDi adherence was associated with reduced odds for PD (Alcalay et al., 2012). A study showed that pretreatment with a probiotic mixture containing *B. animalis lactis, L. rhamnosus GG*, and *L. acidophilus* has neuroprotective effects in PD models. Possibility because of butyrate can induce the BDNF and glial cell line-derived neurotrophic factor (GDNF) upregulated, and monoamine-oxidase was inhibited in the brain. Furthermore, probiotics mixture pretreatment can reduce DA neurons loss, increase the level of DA and reduce the activity of inflammatory cells of brain (Srivastav et al., 2019).

#### Prebiotics

Butyrate produced from bacteria is likely an interesting candidate for PD treatment. Butyrate can induce Atg5- and PI3K/Akt/mTOR-related autophagy way to cause α-syn degradation in a pesticide-induced PD rat model. The abundance of butyrate-producing bacteria elevated in the gut can prevent intestinal barrier dysfunction and increase striatal DA levels (Qiao et al., 2020).

#### Fecal Microbiota Transplantation (FMT)

FMT has a 1700-year history and was proposed to treat human GI diseases (Zhang et al., 2012). Currently, there are multiple ways to modulate gut microbiota, including antibiotics, probiotics, and prebiotics. Moreover, FMT remains an effective method to restore the gut microbiota ecosystem. FMT including screening for a specific microbial population, homogenizing, filtering, and resuspending stool samples, followed by colonoscopy, enema, orogastric tube, or oral delivery in the form of capsules containing lyophilized material (Biagi et al., 2013). Besides PD, FMT has been used to treat various diseases, such as Irritable bowel syndrome (IBS), type 2 diabetes, ulcerative colitis, and neurodegenerative diseases (Glass et al., 2010). Patients with PD often suffer from changes in GI motility. For example, chronic or idiopathic constipation is often found as a co-comorbid condition in PD patients and is associated with colonic and anorectal dyskinesias (Abbott et al., 2007; Mertsalmi et al., 2017; Yu et al., 2018). Several studies have shown that FMT is beneficial for the treatment of constipation in PD and can also significantly improve the non-GI symptoms of patients with neurological diseases (Sun et al., 2018; Huang et al., 2019). The discovery of the gut microbiota regulatory mechanism of PD pathogenesis has been highly valued (Borody and Khoruts, 2012; Cryan and Dinan, 2012). The proposed approach to evaluate FMT as a potential treatment for PD is primarily to assess direct communication of the vagus nerve, changes in neurotransmitter metabolites, activation of immune responses, and production of neuroactive metabolites as well as neurotoxins (Aroniadis and Brandt, 2013). A recent study using a PD mouse model as a recipient found that fecal transplantation from PD patients exacerbates dyskinesias and is associated with a decrease in *Lachnospiraceae* and *Ruminococceae*, which is a genus significantly reduced from PD patients’ stool samples (Keshavarzian et al., 2015). Besides, compared with healthy controls, FMT from PD patients may exacerbate α-syn-related motor dysfunction in α-syn overexpressing mice (Sampson et al., 2016). When transferring the gut microbiota from PD mice to normal mice, striatal neurotransmitter decreasing and motor impairment can be observed in normal mice. In fecal samples of PD, several changes can be observed: fecal SCFAs concentrations were significantly increased, the number of bacteria *Proteobacteria*, *Turicibacterales* and *Enterobacteriales* increased, while *Firmicutes* and *Clostridiales* decreased. FMT can suppress the TLR4/TNF-α signaling pathway which is involved in inflammation of the gut and brain. Finally, FMT administration can improve the gut dysbiosis, decrease fecal SCFAs concentration, increase DA and 5-HT levels, reduce activation of microglia and astrocytes in the substantia nigra, restore motor impairment of PD (Sun et al., 2018). Several clinical cases of PD have shown that FMT treatment can reduce symptoms of co-morbid GI, including bowel disorders, constipation, and ulcerative colitis. Compared to the traditional PD treatment methods mentioned in table 1, FMT has fewer side effects. In future, FMT treatment may also help relieve several non-GI comorbid disorders and provide additional support for the association between gut microbiota and PD. Possible microbiota-targeted intervention strategies can improve health status and prevent PD in the near future.

## Conclusion

The most possible conclusion about the connection between gut microbiota and PD is that: The GI dysfunction could be found in the early stage of PD, α-syn was found in both the gut and brain. The gut disorder exacerbates α-syn deposition and will aggravate neurodegeneration. α-syn deposition may start in the ENS of PD, then accumulate and transfer to the CNS *via* a trans-synaptic cell-to-cell transmission (Lionnet et al., 2018). The imbalance of the gut tract shows a pro-inflammatory environment, the number of the pathogen was elevated, the permeability of the intestinal epithelial barrier also increased. The inflammatory signals could be transferred to the brain through the gut-brain axis and cause brain & behavior dysfunction.

Gut microbiota has been shown as the potential modulator of human health. They play an important role in the intestine system and brain function. Current studies indicate that modify gut microbiota composition can affect brain neurochemistry *via* neural, immune and endocrine. Through antibiotics, probiotics, prebiotics or FMT approach could restore the gut ecosystem and improve brain functions. In the future, more new GI biomarkers need to discovery and the mechanism of specific bacteria through which pathway effect on the host system needs to be clarified.

## Author Contributions

JL contributed in manuscript writing. FX and ZN critically reviewed the manuscript. LS supervised the whole process and reviewed the manuscript. All authors contributed to the article and approved the submitted version.

## Funding

This research was financially supported by the National Natural Science Foundation of China (81773616), Shanghai Excellent Technology Leader Program (17XD1423200), Shanghai Education Development Project: Industry-University-Research Practice (A3-2601-20-311001-11), and the Natural Science Foundation of Shanghai (20ZR1424600).

## Conflict of Interest

The authors declare that the research was conducted in the absence of any commercial or financial relationships that could be construed as a potential conflict of interest.
